# Magnitude of sexual and reproductive health communication between parents and their adolescents: Evidence from Osun State, Nigeria

**DOI:** 10.1371/journal.pgph.0004034

**Published:** 2025-02-10

**Authors:** Olayinka Oluwabusola Bamidele, Agnes Aderinola Oyeniran, Olukemi Adedayo Sabageh, Esther Olufunmilayo Asekun-Olarinmoye

**Affiliations:** 1 Department of Community Medicine, UNIOSUN Teaching Hospital, Osogbo, Nigeria; 2 Department of Health Promotion and Education, College of Medicine, University College Hospital, Ibadan, Nigeria; 3 Department of Community Medicine, College of Health Sciences, Osun State University, Nigeria; Universidad de Sonora, MEXICO

## Abstract

This study evaluated sexual and reproductive health communication (SRHC) between dyads of parents and their in-school adolescents in urban and rural areas of Osun State, Nigeria. A descriptive cross-sectional design was used to determine and compare the prevalence, pattern, level, triggers, and methods of SRHC between 625 parents and their in-school adolescents. Respondents were recruited using the multistage sampling technique, and data was collected using the mixed-method approach. Quantitative data was collected using a semi-structured questionnaire and analysed using chi-square and logistic regression tests, while the qualitative data was collected using a focus group discussion guide and analysed thematically. About 56% of parents (53% rural, 58% urban) and 69% of adolescents (68% rural, 71% urban) reported ever having SRHC. The level of SRHC was 31% for adolescents, with no significant difference by type of residence (36% rural, 33% urban, p=0.324) while for parents it was 59%, with a statistically significant difference by the type of residence (52% rural, 66% urban; p<0.01). A two times higher odds of having a good level of SRHC with their parents was observed among rural female vs. rural male adolescents (OR = 1.9, 95% CI: 1.053 - 3.471, p = 0.033) and among urban early vs. urban late adolescents (OR = 2.2, 95% CI: 1.215 - 4.048, p = 0.009). The odds of having a good level of SRHC were three times higher among mothers (OR: 3.4, 95% CI: 1.409 – 8.422, *P* < 0.007) in urban areas compared to fathers in the same area. SRHC occurred between parents and adolescents in Osun State but with inherent disparities by location and gender. Interventions are required to improve the depth, frequency, and clarity of SRHC, particularly with topics related to experiencing sex. Parents, especially fathers, should be educated about the significance of SRHC and be provided with the skills to engage in early, effective, and sustained discussion with both male and female adolescents.

## Introduction

Adolescents are individuals aged 10-19 years in the adolescence stage of life, a transitional stage characterized by developmental, physiological, and behavioural changes during which an individual reaches sexual maturity [1–3]. Their curiosity about all areas of human undertaking is enhanced by physiological changes in their reproductive organs, which serve as a stimulus in their quest to experiment with sex and sexual relationships [4]. Thus, it is critical for them to be guided appropriately during this stage to make positive and informed decisions about their sexual and reproductive health (SRH). A practical approach to achieve this is through sexual and reproductive health communication (SRHC) [5].

SRHC is the process of sending and receiving messages about SRH issues from one entity or group to another through verbal or nonverbal means using mutually understood signs, signals, and behaviour. Providing SRH education to adolescents is known to play a vital role in preventing risky sexual behaviour and its untoward consequences among this target group [5]. Evidence has shown that communication between adolescents and their parents during this crucial period protects adolescents from engaging in risky sexual practices and associated adverse health consequences [6–9].

Despite these documented benefits, providing SRH information to adolescents is quite challenging because it is a culturally sensitive issue, particularly in Africa [10]. Many African societies have well-established traditions about accepted norms concerning sexual activities and reproduction among the young, as well as specific ways through which relevant SRH information and values are communicated [10].

Premarital sexual activities are prevalent among adolescents in Nigeria and have remained so [11–13]. Studies have reported a high level of sexual activity among unmarried adolescents of both sexes with progressively decreasing ages of sexual debut [11–13]. The problem is not that adolescents are sexually active; instead, they are inadequately prepared and guided in developing responsible sexual behaviours [7]. Adolescents form a more significant percentage of the Nigerian population and are a critical asset to the country’s future; hence, they must be protected and safeguarded. The preceding underscores the role of significant others, including parents, in providing SRHC to adolescents.

Whereas previous studies have focused on SRHC among either parents or adolescents, there is a dearth of information on SRHC between parents and their adolescents from the perspective of dyads of parents (fathers and mothers) and adolescents (sons and daughters) and with a rural/urban comparison. In our extensive literature search, the only few available studies found on SRHC among dyads of parents and adolescents were a mixed-method study conducted in Ghana [14], a qualitative survey of mother-daughter dyads conducted in Northern Nigeria (Kano State) [15], and another mother-daughter dyad study carried out in Uganda but without rural/urban comparison [16].

Exploring SRHC within the context of parent-adolescent dyads goes beyond merely assessing the strength of communication but also delving into the accuracy of information shared within a family unit [15]. Therefore, examining SRHC through the framework of parent-adolescent dyads offers a holistic and balanced evaluation of the content, accuracy, and congruence of information exchanged between parents (fathers and mothers) and their adolescents (sons and daughters). Previous research on SRHC between parents and adolescents has been constrained by the limited inclusion of both members of the parent-adolescent dyad. This restricted approach has hindered a comprehensive understanding of the intricate family dynamics involved in SRHC. However, dyadic analyses of SRHC have demonstrated an ability to unveil between and within variations in communication patterns among members of the parent-adolescent dyad [17].

Furthermore, SRHC, from the perspective of the parent-adolescent dyad, provides an opportunity to effectively capture the reciprocity required in the communication process, particularly within the family unit, which is significantly influenced by the area (rural or urban). As a fundamental unit of society, the family plays a pivotal role in shaping adolescents’ sexual health and behaviours [18]. Understanding the communication dynamics within this unit is imperative for tailoring appropriate public health interventions. While communication is a two-way reciprocal process, the sensitivities associated with SRHC within the African context underscore an increased need for mutuality between dyad members. Therefore, this study centered on dyads of parents and adolescents to contribute nuanced insights into the reciprocal nature of SRHC and identify potential areas for intervention within family structures in both rural and urban areas. Specifically, the study evaluated SRHC by determining and comparing the prevalence, pattern, level, triggers, and methods of SRHC between dyads of parents and their in-school adolescents in urban and rural areas of Osun state.

## Materials and methods

### Study design and setting

**Fig 1:**
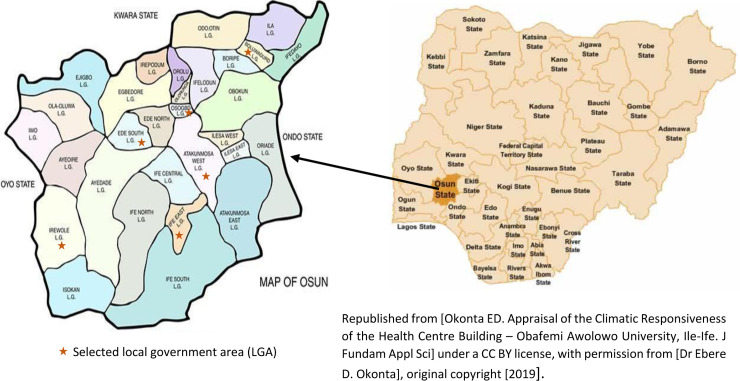
Map of Osun State, Nigeria, showing the selected local government areas [ 21,22**].**

A mixed-method design was used for this study involving a cross-sectional survey for the quantitative aspect and focus group discussions (FGDs) for the qualitative aspect. The study was conducted between1st April and 30th June 2018 among parents and their in-school adolescents in six (Atakumosa West, Boluwaduro, Ede South, Ife East, Irewole and Osogbo) local government areas (LGAs) of Osun State, in southwest Nigeria (Fig 1). Osun State has 30 LGAs, distributed equally among its three senatorial districts (10 LGAs per district). The LGAs are further divided into rural and urban areas [S1 Table]. The State has an estimated population of 4,340,565, of which 23.7% are adolescents, and 55.5% live in rural areas [19,20].

### Participants

The study population was in-school adolescents aged 10–19 years and their parents (biological parents or guardians>18 years) residing in the study sites. A total of 660 parent-adolescent pairs across six LGAs were selected for the quantitative part of the study. The adolescents served as the index participants and were used to recruit their parents into the study with the help of the school authority. The inclusion criteria were adolescents aged 10-19 years attending public or private secondary schools in the State and primarily living with their parents in the past twelve months before the study. Married adolescents and those whose parents were unavailable were excluded from the study. For the qualitative component, eight FGDs were conducted in two LGAs (one rural and urban) among adolescent boys, adolescent girls, male parents, and female parents. Participants for the FGD were chosen by convenience (a non-probability) sampling technique.

### Adolescent participants

A multistage sampling technique was used to select the adolescents, which included a proportionate allocation to determine the number of participants in each of the selected schools and arms (JSS1-SSS3). First, simple random sampling was used to select two LGAs (one rural and one urban) from the three senatorial districts by utilizing the ballot technique. This yielded a total of six LGAs. Second, two secondary schools (one public and one private) were selected from each of the LGAs using simple random sampling by the ballot method. These schools (12) were selected from a list of all registered secondary schools in the six LGAs, as obtained from the state Ministry of Education. Third, simple random sampling was used to select one class from each of the arms in the junior (levels 1-3) and senior (levels 1-3) secondary categories of the respective schools resulting in six classes per school. Proportionate allocation was used to determine the total number of students to be interviewed in each of the selected schools. This was calculated as below:


$$\frac{Number\;of\;students\;in\;the\;selected\;school}{Total\;number\;of\;students\;in\;all\;selected\;schools}\;\;\;\;X\;\;Study\;sample\;size\;\;\;$$ (1)

The total number of students calculated for each school was allocated proportionately across the six classes selected from the junior and senior secondary categories. This allocation was used in determining the number of students to be interviewed in each of the selected classes. It was calculated using the number of eligible students in each class, the total number of eligible students in all the selected classes in each school, and the sample size for each school.


$$\frac{number\;of\;students/class}{Total\;number\;of\;students\;in\;all\;the\;selected\;classes/school}\;\;\;\;X\;\;Sample\;size/school\;$$ (2)

The last stage adopted systematic random sampling to select participants in each of the chosen classes using the class register as the sampling frame. The first participant was selected by simple random sampling using the ballot method. Subsequent participants were selected based on the sampling interval in each class until the sample size was achieved. The adolescents participated in the study at their respective schools.

### Parent participants

The parents participated in the study within the household settings. Both parents were eligible to participate in the survey, but only one was allowed. Where both parents were willing to participate, a simple ballot was used to select the participating parent randomly.

### Sample size

The sample size was determined using the formula to compare two proportions [23]. The prevalence of SRHC among adolescents in the urban area was 63.4% [24], and an assumed prevalence of 50% was used for the rural area to obtain a minimum sample size of 287. After adjusting for a 10% non-response rate, a sample size of 660 parent-adolescent pairs was finally used.

### Instrument and variables

The data was collected via pre-tested, semi-structured questionnaires (adapted from previous studies [7,14] with separate questionnaires for adolescents and their parents) and an FGD guide. The questionnaire was modified without obtaining permission from its original owner. Both tools were translated into the local language, Yoruba, for ease of comprehension. Content validity of the tools was ensured through translation-back translation (English-Yoruba-English) by separate translators. The reliability test, which measured the internal consistency of the adolescents’ and parents’ questionnaires, yielded an overall Cronbach’s alpha of 0.98 for each group of participants.

The questionnaires were used to elicit information on participants’ demographic characteristics, prevalence and pattern of SRHC, and SRH topics discussed. Sociodemographic characteristics include the following:

1. Age- Adolescents (10-14 years, 15 -19 years): Parents (40 and below; 41 – 60, Above 60)
2. Gender – Male, female
3. Religion – Christianity, Islam, Traditional
4. Class – (for adolescents) JSS 1-3, SSS 1-3
5. Birth Order - 1st, 2nd-5th, 6^th^ -9^th^
6. living with - (for adolescents) Both parents, mother only, female guardian, male guardian, father only
7. Educational Level (for parents) – Primary and below, Secondary, Tertiary
8. Occupation (for parents) – Business/trader, Civil servants, farmer, artisans
9. Income (for parents) - < N20,000, N 20,000 – N 50,000, > N 50,000
10. Marital status (for parents) – single, married, divorced, widowed
11. Marriage type (for parents) – monogamy, polygamy
12. Planned marriage (for parents) – yes, no.

Prevalence was measured based on the singular question of “have you ever discussed SRH issues with your child or have your parents discussed SRH issues with you”. The SRH topics were measured through 20 distinct items and categorized into three domains. The biological/developmental domain had five items (physical development, menstruation/ wet dreams, puberty, masturbation, and reproduction/having babies), sexual risk prevention or safety domain had eight items (prevention of STI, prevention of HIV/AIDS, condom & other contraceptives use, abstaining from sex till marriage, pregnancy, abortion, consequences of premarital sex, and substance use), and experiencing sex domain had seven items (sexual feeling, when to commence sexual intercourse, choosing sexual partners, how to handle sexual pressure, homosexuality, pornography, and rape) [14]. Discussion of SRH topics was measured on a 4-point Likert scale (0 = never, 1 = once, 2 = a few times, and 3 = often) and was used to rate the level of communication, which was scored and graded as poor (≤ 30) and good (>30). For the adolescents, the mean SRHC score for father and mother communication was used to rate the level of communication. The main purpose of the FGD as a qualitative tool was to aid the quantitative aspect to further explore in depth knowledge and also to triangulate findings from the quantitative aspect.

### Data collection procedure

The data was collected by trained research assistants (RAs) who administered the questionnaire to the adolescents at their school. In-school adolescents served as leads for selecting parents with the help of the school authority. After that, the RAs contacted the parents and administered the questionnaire to the parents in their homes. The qualitative part of the study was facilitated by the researcher, who was assisted by the trained RAs as note-takers. Eight FGDs were conducted in two LGAs (one rural and one urban), separately among adolescent boys, adolescent girls, male parents, and female parents in each LGA to ensure homogeneity. The FGDs were double audio-recorded, and each lasted 45 minutes to one hour.

### Statistical analysis

Of the 660 parent-adolescent pairs responses received, 35 pairs were excluded due to incomplete information. Hence, data from 625 parent-adolescent pairs were included in this analysis. Statistical analysis was performed using Statistical Product and Service Solutions (SPSS) version 21, with the significance level set at p ≤0.05. Univariate analysis was done to generate frequencies and percentages, and Chi-square (χ2) test statistics was utilized to assess association and compare differences between two categorical variables. Continuous variables were presented as mean ± standard deviation. Logistic regression was used to estimate the odds ratio at 95% confidence intervals for significant variables at Chi-square to estimate the magnitude and direction of the relationship. e.g., SRHC level of adolescents/parents with sociodemographic factors that are significant at chi-square. The audio-recorded FGD sessions were transcribed verbatim, and a report of each was computed. These reports were analysed thematically through group coding of similar responses, and quotes that buttressed the quantitative component of the study were integrated into the findings.

### Ethical considerations

The study was conducted in accordance with the Declaration of Helsinki. It was approved by the Ethics Review Committee of the Osun State Ministry of Health on August 6, 2017 (Approval no. OSHREC/PRS/569T/143). Official permission to carry out the study was obtained from the Osun State Ministry of Education and the principals of the selected schools. Informed written consent was obtained from participants ≥ 18 years and assent from those < 18 years after obtaining written consent from parents/guardian; parents also signed consent forms before completing the questionnaires. Anonymity and confidentiality of responses were assured, and participants had the right to refuse or withdraw participation at any time during the study.

## Results

### Comparison of socio-demographic characteristics by type of residence

Table 1 shows the socio-demographic characteristics of the participants. Adolescents’ mean age was 14.23 ± 2.0 years, 14.19 ± 2.1 years in the rural area, and 14.40 ± 2.0 in the urban area. There were more females (58.4%) than males (41.6%), and this was comparable for the type of residence. More than half (54.9%; 52.5% rural; 57.1% urban) were in junior secondary school. Most adolescents live with both parents (62.4%; 61.1% rural; 63.7% urban). There were significant differences in religion and birth order. (p<0.001). A more substantial percentage of the parents were between 41 and 60 years (54.6%). However, more parents in the rural area were 40 years and below (48.5%), with a significantly lower percentage of urban parents (33.9%) in this age category (p=<0.001). More female parents (68%) participated in the study than male parents (32%) in rural and urban residences. There were significant differences in parental age p=<0.001, income p=<0.001, religion p=0.001 marital status p=<0.001, type of marriage p=0.030, and whether the marriage was planned p=0.020 across the type of residence.

**Table 1:** Socio-demographic characteristics of respondents according to their residence.

Variables		Adolescents		Statistics		Parents		Statistics
	Total N = 625	Rural n = 303	Urban n = 322		Total N =625	Rural n = 303	Urban n = 322	
**Gender**				χ^2^ = 3.163				χ^2^ = 1.903
Male	260 (41.6)	137 (45.2)	123 (38.2)	df = 1	200 (32.0)	105 (34.7)	95 (27.5)	df = 1
Female	365 (58.4)	166 (54.8)	199 (61.8)	p = 0.075	425 (68.0)	198 (65.3)	227 (72.5)	p = 0.168
**Religion**								
Christianity	345 (55.2)	190 (62.7)	155 (48.1)	χ^2^ = 17.110	419 (67.0)	181 (59.7)	238 (73.9)	χ^2^ = 14.392
Islam	278 (44.5)	111 (36.6)	167 (51.9)	df = 2	202 (32.4)	120 (39.6)	82 (25.5)	df = 2
Traditional	2 (0.3)	2 (0.7)	0 (0.0)	p = <0.001*	4 (0.6	2 (0.7)	2 (0.6)	p = 0.001*
**Class**				χ^2^ = 1.373				
JSS I – JSS 3	343 (54.9)	159 (52.5)	184 (57.1)	df = 1				
SSS1 - SSS 3	282 (45.1)	144 (47.5)	138 (42.9)	p = 0.241				
**Birth Order**				χ^2^ = 9.77				
1	158 (28.4)	60 (22.4)	98 (33.9)	df = 2				
2 -5	372 (66.8)	196 (73.1)	176 (60.9)	p = 0.008*				
6 -9	27 (4.8)	12 (4.5)	15 (5.2)					
**Lives with**								
Both Parents	390 (62.4)	185 (61.1)	205 (63.7)					
Mother only	129 (20.6)	66 (21.8)	63 (19.6)	χ^2^ = 4.460				
Female guardian	59 (9.4)	24 (7.9)	35 (10.9)	df = 4				
Male guardian	28 (4.5)	16 (5.3)	12 (3.7)	p = 0.347				
Father only	19 (3.0)	12 (4.0)	7 (2.2)					
**Age group in years**				χ^2^ = 2.048				
10 -14	353 (56.5)	180 (59.4)	173 (53.7)	df = 1				
15 -19	272 (43.5)	123 (40.6)	149 (46.3)	p = 0.152	256 (41.0)	147 (48.5)	109 (33.9)	χ^2^ = 29.861
*Mean age*		14.19 ± 2.1	14.40 ± 2.0	t(527) =-1.045, p=0.161				
40 and below					341 (54.6)	134 (44.2)	207 (64.3)	df = 2
41–60					28 (4.5)	22 (7.3)	6 (1.9)	p = <0.001*
Above 60								t(618)=
*Mean Age*		43.07 ±12.4	44.26 ± 8.4	t(618) =-1.269, p=0.205				
**Educational Level**								
Primary and below					101 (16.1)	41 (13.5)	60 (18.6)	χ^2^ = 3.007
Secondary					251 (40.2)	126 (41.6)	125 (38.8)	df = 2
Tertiary					273 (43.7)	136 (44.9)	137 (42.5)	p = 0.222
**Occupation**								
Business/trader					348 (55.7)	156 (51.5)	192 (59.6)	
Civil servant					183 (29.3)	91 (30.0)	92 (28.6)	χ^2^ = 6.686
Farmer					61 (9.8)	37 (12.2)	24 (7.5)	df = 3
Artisans					33 (5.2)	19 (6.3)	14 (4.4)	p = 0.083
**Income**								
< ₦ 20,000					303 (48.5)	186 (61.4)	117 (36.3)	χ2 = 39.572
₦ 20,000 – ₦ 50,000					251 (40.2)	89 (29.4)	162 (50.3)	df = 2
> ₦ 50,000					71 (11.4)	28 (9.2)	43 (13.4)	p = <0.001*
**Marital status**								
Single					42 (6.7)	34 (11.2)	8 (2.5)	
Married					534 (85.4)	247 (81.5)	287 (89.1)	χ2 = 19.118
Divorced					30 (12.7)	13 (4.3)	17 (5.3)	df = 3
Widowed					19 (3.0)	9 (3.0)	10 (3.1)	p = <0.001*
**Marriage Type#(n=583)**								χ^2^ = 4.721
Monogamy					481 (82.5)	212 (78.8)	269 (85.7)	df = 1
Polygamy					102 (17.5)	57 (21.2)	45 (14.3)	p = 0.030*
**Planned marriage#**								χ^2^ = 5.391
Yes					478 (82.0)	228 (86.0)	250 (78.6)	df = 1
No					105 (18.0)	37 (14.0)	68 (21.4)	p = 0.020*

*Statistically significant, JSS 1–3 - Junior Secondary School 1,2,3, SSS 1-3-Senior Secondary School 1,2,3. # married only, numbers in parenthesis are proportions.

### Comparison of the prevalence and pattern of SRHC by type of residence

The prevalence of SRHC was measured using a single dichotomous variable. Have you ever discussed SRH issues with your adolescent/parents (Yes/No)? About 70% of adolescents and 55.5% of parents responded affirmatively (Table 2). In urban areas, 70.8% of adolescents and 57.8% of parents reported discussing SRH issues, compared to 67.7% of adolescents and 53.1% of parents in rural areas. In the FGDs, 67.0% (20 out of 29) of adolescents and 58.6% (17 out of 29) of parents answered yes to the same question.

**Table 2:** Prevalence of SRHC according to respondents by their residence.

Ever discussed issues about SRH	Residence			Statistics
	Rural n = 303	Urban n = 325	Total N = 625	
**Adolescents** Yes	205 (67.7)	228 (70.8)	433 (69.3)	χ ^2^ = 0.728 df = 1 p = 0.393
**Parents** Yes	161 (53.1)	186 (57.8)	347 (55.5)	χ ^2^ = 1.354 df = 1 p = 0.245

Table 3 gives an overview of parents’ reasons for not discussing SRH issues with their adolescents. Overall, 44% of parents never discussed SRH with their adolescents. Reasons adduced by parents who had never had SRHC with their adolescents (278/625, 44%) were mainly that their adolescents were not mature enough for such discussion (74.1%). However, more urban parents (83.8%) significantly proffered this reason than the rural parents (64.8%), p=<0.001. Other reasons were the perception that it makes the child promiscuous (rural, 12.7%; urban, 7.4%), p=0.146, initiating the discussion is embarrassing (rural, 9.9%; urban, 13.2%), p=0.378, and the child will learn as he/she grows (rural, 11.2%; urban, 13.3%), p=0.617. Furthermore, some fathers stated that it is the mother’s duty (7.9%), and this perception was statistically significant by the type of residence (rural, 2.8%; urban, 13.2%) (p=0.001) [Table 3].

**Table 3:** Parents’ reasons for not discussing SRH issues with their adolescents.

Variables^	Residence			P-value
	Rural n = 142	Urban n = 136	Total N = 278	
Child is not mature enough for such a discussion	92 (64.8)	114 (83.8)	206 (74.1)	<0.001*
May make a child promiscuous	18 (12.7)	10 (7.4)	28 (10.1)	0.146
Child will learn as he/she grows	16 (11.2)	18 (13.3)	34 (12.3)	0.617
Consider it odd/sensitive/embarrassing	14 (9.9)	18 (13.2)	32 (11.5)	0.378
It is not good/ethically right/necessary	8 (5.6)	8 (5.9)	16 (5.8)	0.929
Busy/No time	6 (4.2)	12 (8.8)	18 (6.5)	0.119
Duty of the mother^#^	4 (2.8)	18 (13.2)	22 (7.9)	0.001*
Child has been taught in school	2 (1.4)	2 (1.5)	4 (1.4)	0.965
Child is not interested	2 (1.4)	2 (1.5)	4 (1.4)	0.965

# Response obtained from only male parents. * Statistically significant, ^ - multiple responses.

The FGD discussants stated that the reasons for the lack of SRHC is because their work schedule does not permit it, and they never benefitted from such discussions as young people. One of the discussants emphasized this perception about SRHC, which is indicative of an authoritarian parenting style:

R: *I don’t know the meaning, I don’t understand (all laughed). The reason why I don’t understand is that no parent ever discussed such with me before about sex and reproduction. The only thing I used to tell my children is to make sure they pursue their academics to the peak, and if you will learn a trade and make sure you have good work, I tell both males and females. That is what I do tell them most of the time*. _ **Male parent, rural area.**

The majority of the adolescents had reached the stage of adolescence before SRHC was initiated. There was no significant difference in the reported age of SRHC initiation by adolescents (72.2%, 77.2%, p = 0.232) and their parents (85.1%, 81.2%, p = 0.334) in the rural and urban areas, respectively. Among the adolescents, the frequency (p=0.088) and duration of SRHC (p= 0.190) were not significantly different by the type of residence. However, this difference (frequency (p = 0.002) and duration of SRHC (p = < 0.001) was statistically significant among the parents [Table 4]. A similar view on SRHC initiation was shared by the FGD discussants, with 15 of the 27 adolescents and 20 out of 23 parents in both areas reporting SRHC between 10 and 19 years. However, some of the parents stated that SRHC should begin before age ten due to early puberty onset:

**Table 4:** Pattern of SRHC according to adolescents and parents by their residence.

Variables		Adolescents				Parents		
	Total	Rural	Urban	Statistics	Total	Rural	Urban	Statistics
	N = 433	n = 205	n = 228		N = 347	n = 161	n = 186	
**Frequency of SRH discussion**								
Daily	146 (33.7)	70 (34.1)	76 (33.3)	χ ^2^ = 6.542	61 (17.6)	31 (19.3)	30(16.1)	χ ^2^ = 14.567
Weekly	94 (21.7)	53 (25.9)	41 (18.0)	df = 3	60 (17.3)	39 (24.2)	21 (11.3)	df = 3
Monthly	105 (24.3)	49 (23.9)	56 (24.6)	p = 0.088	48 (13.8)	24 (14.9)	24 (12.9)	p = 0.002
No specific interval	88 (20.3)	33 (16.1)	55 (24.1)		152 (43.8)	67 (31.7)	111 (59.7)	
**Average length of discussion**								
Less than 10 minutes	140 (32.3)	70 (34.1)	70 (30.6)	χ ^2^ = 4.763	54 (15.5)	26 (16.1)	28(15.1)	χ ^2^ = 23.655
10 – 30 minutes	130 (30.1)	61 (29.8)	69 (30.3)	df = 3	91 (26.2)	49 (30.4)	42 (22.6)	df = 3
.> 30 minutes	91 (21.0)	35 (17.1)	56 (24.6)	p = 0.190	46 (13.3)	33 (20.5)	13 (7.0)	p = < 0.001*
Not specific	72 (16.6)	39 (19.0)	33 (14.5)		156 (45.0)	53 (32.9)	103 (55.4)	
**Age at initiation of discussion**								
Less than 10 years	109 (25.2)	57 (27.8)	52 (22.8)	χ ^2^ = 1.431	59 (17.0)	24 (14.9)	35 (18.8)	χ ^2^ =0.935
10 – 19 years	324 (74.8)	148 (72.2)	176 (77.2)	df = 1	288 (83.0)	137 (85.1)	151 (81.2)	df = 1
				p = 0.232				p = 0.334

*Statistically significant.

R: *Because the world has changed. In those days, before a girl could reach puberty, it would be up to 14 years and above, but nowadays, a girl can reach puberty at ten years of age, even, I can say, nine years. Hence, let’s say ten years of age for both males and females*. _ **Male parent, rural area.**

R: …*About five years, because nowadays, a child of 2, 3 years of age has started school already and whatever he has been taught he would not forget*. _ **Female parent, rural area**

### Comparison of triggers and methods of SRHC by type of residence

**Fig 2:**
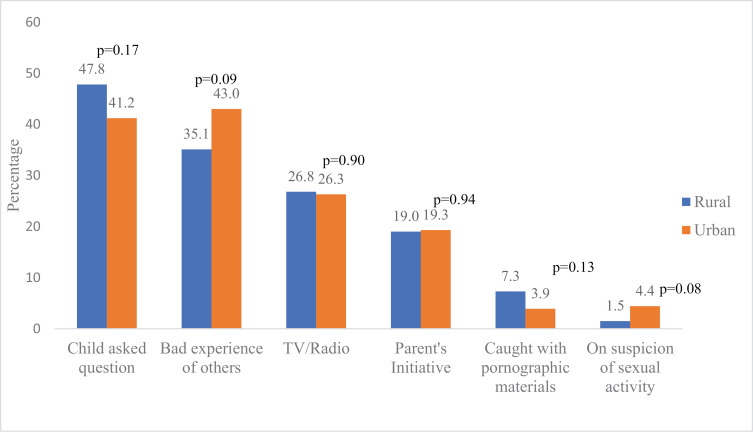
Triggers of SRHC among adolescents according to their residence.

**Fig 3:**
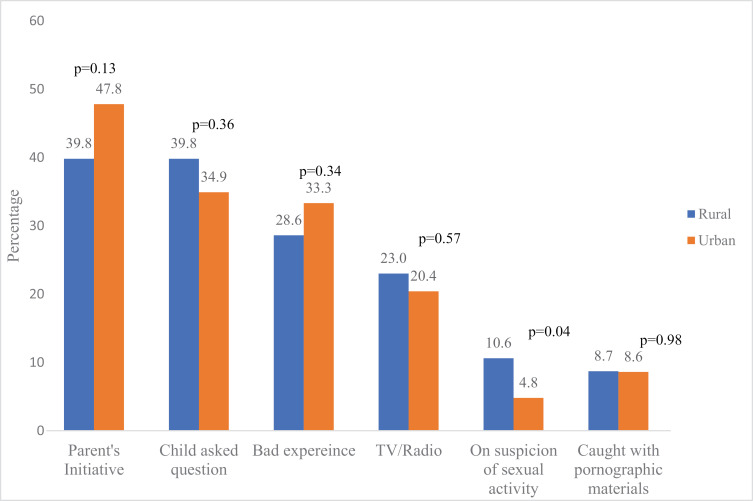
Triggers of SRHC among parents according to their residence.

The triggers of SRHC among adolescents were comparable in rural and urban areas (Fig 2). Asking questions by adolescents (47.8%) ranked highest among respondents from rural areas, while bad experiences of other people (43.0%) ranked highest among urban adolescents. Parental initiative was the most reported trigger of SRHC for parents in both rural (39.8%) and urban (47.8%) (Fig 3). There was no significant difference in the reported triggers of SRHC except for that on suspicion of sexual activity: more parents (17, 10.6%) in the rural areas significantly had SRHC based on suspicion of sexual activity compared to their urban (9, 4.8%) counterparts (p=0.04) (Fig 3).

**Fig 4:**
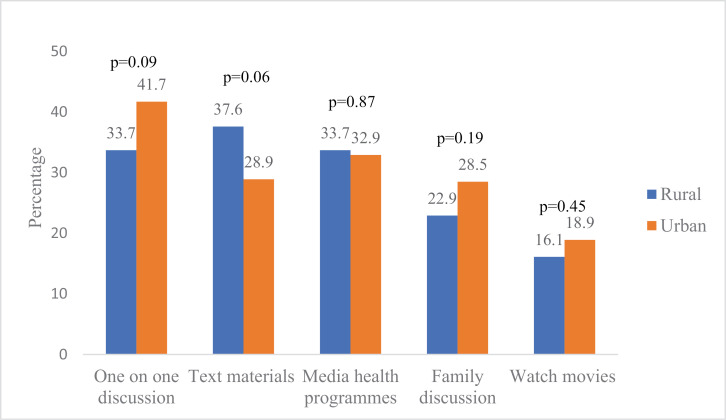
Comparison of methods adopted for SRHC among adolescents by type of residence.

**Fig 5:**
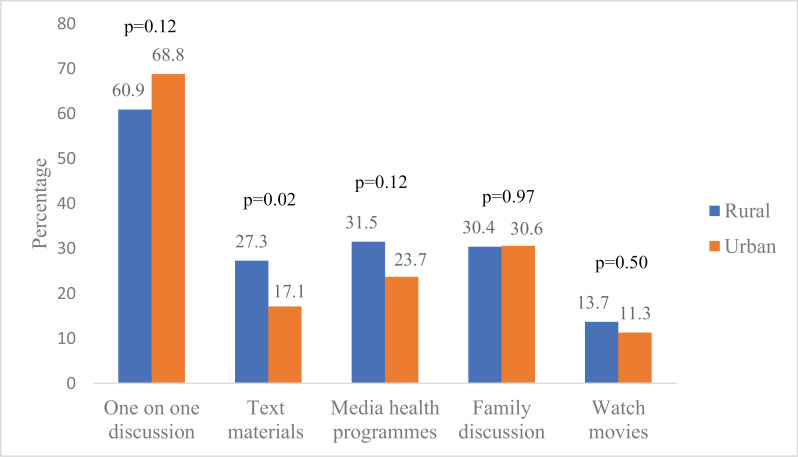
Comparison of methods adopted for SRHC among parents by type of residence.

Similar methods of SRHC were adopted in the two study areas. The primary method reported by adolescents in the rural area was the giving of text materials by parents (37.6%), while one-on-one discussion ranked highest among their urban counterparts (41.7%) (Fig 4). One-on-one discussion ranked highest among methods adopted for SRHC among parents in rural (60.9%) and urban (68.8%) areas. There was no significant difference in the reported methods of SRHC adopted except for the use of text materials: more parents in the rural regions significantly adopted the use of text materials for SRHC compared to their urban counterparts (p=0.02) (Fig 5).

### Comparison of SRH topic discussed by type of residence

SRH topics discussed by parents and their adolescents were measured using 20 topics, categorized into three domains: biological/developmental, sexual risk prevention, and experiencing sex. In addition, adolescents were asked to specify which of their parents, mother/father, discussed any of the topics with them. Overall, more parents in the urban areas reported discussing SRH topics with their adolescents than those in the rural areas. There was a statistically significant difference in the proportion of parents in the urban and rural who discussed 11 of the 20 SRH topics (Reproduction/having babies (80.6%,67.7%, p-0.006; prevention of STIs (88.7%,74.5%,p-0.001; prevention of HIV/AIDS (88.2%,78.3%, p-0.013; abstaining from sex till marriage (91.4%,83.9%, p-0.032; pregnancy (81.7%,70.2%, p-0.012; abortion (70.0%,59.0%,p-0.019); consequences of premarital sex (86.0%,71.4%,p-0.001); sexual feeling (69.4%,46.4%,p-0.044); when to start sexual intercourse (73.7%,61.5%, p-0.015); choosing sexual partners (72.6%,62.1%,p-0.038); rape (72.6%,59.6%, p-0.011) [Table 5].

**Table 5:** Topics discussed as reported by adolescents and parents according to their residence.

SRH Topics		Parent						Adolescents					
		Total	Rural	Urban		Mother					Father		
	p-value				P-value	Total	Rural		Urban	p-value	Total	Rural	Urban
**Biological/developmental**													
Physical Development	0.323	316 (91.1)	144 (89.4)	172 (92.5)	0.544	333 (76.9)	155 (75.6)		178 (78.1)	0.009*	301 (69.5)	155 (75.6)	146 (64.0)
Menstruation/ Wet dreams	0.140	299 (86.2)	134 (83.2)	165 (88.7)	0.020*	336 (77.6)	149 (72.7)		187 (82.0)	0.068	257 (59.4)	131 (63.9)	126 (55.3)
Puberty	0.168	318 (91.6)	144 (89.4)	174 (93.5)	0.036*	336 (77.6)	150 (73.2)		186 (81.6)	0.013*	300 (69.3)	154 (75.1)	146 (64.0)
Masturbation	0.160	152 (43.8)	77 (47.8)	75 (40.3)	0.738	236 (54.5)	110 (53.7)		126 (55.3)	0.015*	156 (36.0)	86 (42.0)	70 (30.7)
Reproduction/having babies	0.006*	259 (74.6)	109 (67.7)	150 (80.6)	0.613	261 (60.3)	121 (59.0)		140 (61.4)	0.103	206 (47.6)	106 (51.7)	100 (43.9)
**Sexual risk prevention**													
Prevention of STIs	0.001*	285 (82.1)	120 (74.5)	165 (88.7)	0.028*	299 (69.1)	131 (63.9)		168 (73.7)	0.138	278 (64.2)	139 (67.8)	139 (61.0)
Prevention of HIV/AIDS	0.013*	290 (83.6)	126 (78.3)	164 (88.2)	0.488	311 (71.8)	144 (70.2)		167 (73.2)	0.006*	282 (65.1)	147 (71.7)	135 (59.2)
Abstaining from sex till marriage	0.032*	305 (87.9)	135 (83.9)	170 (91.4)	0.047*	305 (70.4)	135 (65.9)		170 (74.6)	0.001*	299 (69.1)	157 (76.6)	142 (62.3)
Condom & other contraceptive use	0.208	185 (53.3)	80 (49.7)	105 (56.5)	0.808	216 (49.9)	101 (49.3)		115 (50.4)	0.066	187 (43.2)	98 (47.8)	89 (39.0)
Pregnancy	0.012*	265 (76.4)	113 (70.2)	152 (81.7)	0.061	263 (60.7)	115 (56.1)		148 (64.9)	0.041*	212 (49.0)	111 (54.1)	101 (44.3)
Abortion	0.019*	227 (65.4)	95 (59.0)	132 (71.0)	0.577	230 (53.1)	106 (51.7)		124 (54.4)	0.065	189 (43.6)	99 (48.3)	90 (39.5)
Consequences of premarital sex	0.001*	275 (79.3)	115 (71.4)	160 (86.0)	0.744	269 (62.1)	129 (62.9)		140 (61.4)	<0.001*	244 (56.4)	134 (65.4)	110 (48.2)
Substance use	0.472	171 (49.3)	76 (47.2)	95 (51.1)	0.887	236 (54.5)	111 (54.1)		125 (54.8)	<0.001*	210 (48.5)	119 (58.0)	91 (39.9)
**Experiencing sex**													
Sexual feeling	0.044*	224 (64.6)	95 (46.4)	129 (69.4)	0.738	236 (54.5)	110 (53.7)		126 (55.3)	0.010*	198 (45.7)	107 (52.2)	91 (39.9)
When to start sexual intercourse	0.015*	236 (68.0)	99 (61.5)	137 (73.7)	0.699	245 (56.6)	114 (55.6)		131 (57.5)	0.015*	212 (49.0)	113 (55.1)	99 (43.4)
Choosing sexual partners	0.038*	235 (67.7)	100 (62.1)	135 (72.6)	0.453	222 (51.3)	109 (53.2)		113 (49.6)	0.001*	193 (44.6)	108 (52.7)	85 (37.3)
How to handle sexual pressure	0.181	222 (64.0)	97 (60.2)	125 (67.2)	0.324	230 (53.1)	114 (55.6)		116 (50.9)	0.003*	200 (46.2)	110 (53.7)	90 (39.5)
Homosexuality	0.207	147 (42.4)	74 (46.0)	73 (39.2)	0.453	222 (51.3)	109 (53.2)		113 (49.6)	0.073	175 (40.4)	92 (44.9)	83 (36.4)
Rape	0.011*	231 (66.6)	96 (59.6)	135 (72.6)	0.808	216 (49.9)	101 (49.3)		115 (50.4)	0.054	184 (42.5)	97 (47.3)	87 (38.2)
Pornography	0.514	201 (57.9)	90 (55.9)	111 (59.7)	0.054	226 (52.2)	117 (57.1)		109 (47.8)	0.002*	174 (40.2)	98 (47.8)	76 (33.3)

*Statistically significant.

From the adolescents’ perspective, urban mothers discussed 15 SRH topics more than their rural counterparts, while rural mothers discussed five SRH topics more than their urban counterparts. These five topics were choosing sexual partners, handling sexual pressure, consequences of premarital sex, homosexuality, and pornography. However, only discussion on menstruation/wet dreams (82.0%,72.7%, p-0.020), puberty (81.6%,73.2%, p-0.036), prevention of STI (73.7%,63.9%,p-0.028)and abstaining from sex till marriage (74.6%,65.9%, p-0.047), differed significantly between urban and rural mothers. By contrast, the adolescents reported more SRH discussion across all 20 topics by rural fathers compared to their urban counterparts. This difference was statistically significant across 13 topics [physical development (64.0%, 75.6%, p-0.009); puberty (64.0%, 75.1%, p-0.013); masturbation (30.7%, 42.0%, p-0.015); prevention of HIV/AIDS (59.2%, 71.7%, p-0.006); abstaining from sex till marriage (62.3%, 76.6%, p-0.001); pregnancy (44.3%, 54.1%, p-0.041); consequences of premarital sex (48.2%, 65.4%, p-<0.001); substance abuse (39.9%, 58.0%, p-<0.001); sexual feeling (39.9%, 52.2%, p-0.010); when to start sexual intercourse (43.4%, 55.1%, p-0.013); choosing sexual partners (37.3%, 52.7%,p-0.001); how to handle sexual pressure (39.5%, 53.7%, p-0.003); pornography (33.3%,47.8%, p-0.002) except for seven topics (masturbation, reproduction/having babies, prevention of STIs, condom and other contraceptive use, abortion, homosexuality, and rape) [Table 5].

In the FGD sessions, both adolescents and parents in rural and urban areas stated that menstruation is a commonly discussed SRH topic between them. Parents and urban adolescents also mentioned puberty, while parents and rural adolescents said prevention of STI and HIV/AIDS. One of the parents described the topics she has discussed with her adolescents, which cuts across the three domains:

R: *I called my children, my firstborn, that with your level of maturity, if you are not careful and face your studies, if you have a secret girlfriend and you are having sex secretly, and she gets pregnant, that shows that you can take care of the responsibility by yourself, don’t do that, and the one that is female I told her, you have started menstruating, if you allow a man to have sex with you and you get pregnant that means you are ripe and ready for family life*. _ **Female parent, urban area.**

R: *My mom told me that I should not move close to boys, that the boy should not touch my private parts, and I should not have any sex with boys, and when a woman gets pregnant and engages in abortion, that woman can either die or her womb may be destroyed. Also, she said when it’s time for menstruation, they will be telling me everything about when menstruation will come*. _**Adolescent girl, urban area**

### Comparison of the level of SRHC by type of residence

The level of SRHC based on the perspectives of the adolescents and their parents by the type of residence is shown in Table 6. Participants’ level of SRHC was measured by recoding and scoring the 4-point Likert scale (0 = never, 1 = once, 2 = a few times, and 3 = often) used to measure discussion of SRH topics. After scoring, this was graded as poor (≤ 30) and good (>30). For the adolescents, the mean SRHC score for father and mother communication was used to rate the level of SRHC. From the adolescents’ perspective, the level of SRHC between them and their parents was poor (66.7%), with no significant difference between the rural and urban adolescent respondents. Based on the parents’ responses, however, about 60% achieved a good level of SRHC, with statistically significant differences (p-0.006) by type of residence: more parents in the urban areas (66.1%) had a good level of SRHC compared to those in the rural areas (51.6%).

**Table 6:** Respondents’ level of SRHC according to their residence.

Level of SRHC	Total	Rural	Urban	p-value
**Adolescents**	**n=433**	**n=205**	**n=228**	
Poor	289 (66.7)	132 (64.4)	157 (68.9)	χ ^2^ = 0.971
Good	144 (33.3)	73 (35.6)	71 (31.1))	p = 0.324
**Parents**	**n = 347**	**n = 161**	**n = 186**	
Poor	141(40.6)	78(48.4)	63(33.9)	χ ^2^ = 7.601
Good	206(59.4)	83(51.6)	123(66.1)	p = 0.006*

* Statistically significant.

### Comparison of socio-demographic characteristics by level of SRHC

We evaluated the association between participants’ socio-demographic characteristics and level of SRHC by the type of residence [Table 7]. Female adolescents (41.4%) in the rural areas had a significantly higher level of SRHC with their parents than their male counterparts (27.0) (p=0.032). Early adolescents (36.5%) reported a substantially higher SRHC level than late adolescents (20.6%) (p=0.009) in the urban area Among rural parents, income, planned marriage, and type of marriage were significantly associated with the level of SRHC. In contrast, among urban parents, there was a significant relationship between the level of SRHC and age, sex, education, occupation, income, and planned marriage.

**Table 7:** Association of socio-demographic characteristics and level of SRHC by their residence.

Socio - demographics	Level of SRHC(Rural)		Statistics	Level of SRHC(Urban)		Statistics
**Poor**	**Good**		**Poor**	**Good**
**Adolescents**						
**Age group**						
10 – 14	66 (59.5)	45 (40.5)	χ ^2^ = 3.119	80 (63.5)	46 (36.5)	χ ^2^ = 6.885
15 – 19	67 (71.3)	27 (28.7)	p = 0.077	81 (79.4)	21 (20.6)	p = 0.009**
**Sex**						
Male	65 (73.0)	24 (27.0)	χ ^2^ = 4.591	52 (71.2)	21 (28.8)	χ ^2^ = 0.020
Female	68 (58.6)	48 (41.4)	p = 0.032*	109 (70.3)	46 (29.7)	p = 0.888
**Religion**						
Christianity	81 (65.9)	42 (34.1)	LR = 0.281	72 (69.2)	32 (30.8)	χ ^2^ = 0.176
Islam	51 (63.8)	29 (36.2)	p = 0.869	89 (71.8)	35 (28.2)	p = 0.675
Traditional	1 (50.0)	1 (50.0)				
**Birth position**						
1st	25 (56.8)	19 (43.2)	LR=0.542	53 (74.6)	18 (25.4)	LR =1.470
2^nd^ – 5^th^	82 (63.1)	48 (36.9)	p = 0.762	83 (66.9)	41 (33.1)	p = 0.479
6^th^ – 9^th^	5 (62.5)	3 (37.5)		7 (63.6)	4 (36.4)	
**Parents**						
**Age group**						
≤ 40	35 (56.5)	27 (34.5)	LR = 2.637	23 (47.9)	25 (52.1)	LR = 7.686
41 – 60	37 (43.0)	49 (57.0)	p = 0.268	37 (27.8)	96 (72.2)	p = 0.021*
> 60	6 (46.2)	7 (53.8)		3 (60.0)	2 (40.0)	
**Sex**						
Father	28 (59.6)	19 (40.4)	χ ^2^ = 3.291	20 (50.0)	20 (50.0)	χ ^2^ = 5.919
Mother	50 (43.9)	64 (56.1)	p = 0.070	43 (29.5)	103(70.5)	p = 0.015*
**Education**						
Primary and below Secondary	6 (31.6) 36 (56.3)	13 (68.4) 28 (43.8)	χ ^2^ = 3.889 p = 0.143	3 (10.3) 20 (27.4)	26 (89.7) 53 (72.6)	χ ^2^ =16.870 p = 0.001*
Tertiary	36 (46.2)	42 (53.8)		40 (47.6)	44 (52.4)	
**Occupation**						
Business/trader	50 (55.6)	40 (44.4)	LR = 5.525	34 (29.8)	80 (70.2)	χ ^2^ =13.056
Civil servant	17 (35.4)	31 (64.6)	p = 0.137	20 (33.9)	39 (66.1)	p = 0.005*
Farmer	8 (44.4)	10 (55.6)		4 (66.7)	2 (33.3)	
Artisans	3 (60.0)	2 (40.0)		5 (71.4)	2 (28.6)	
**Income**						
< ₦20,000	60 (57.1)	45 (42.9)	LR=11.260	33 (50.0)	33 (50.0)	χ ^2^ = 20.265
₦20,000-₦50,000	15 (37.5)	25 (62.5)	p = 0.004*	16 (17.8)	74 (82.2)	p< 0.001*
> ₦50,000	3 (18.8)	13 (81.3)		14 (46.7)	16 (53.3)	
**Marital Status**						
Single	4 (80.0)	1 (20.0)	LR = 4.886	1 (33.3)	2 (66.7)	χ ^2^ = 4.619
Married	66 (45.8)	78 (54.2)	p = 0.264	55 (32.5)	114 (67.5)	p = 0.266
Divorced	6 (75.0)	2 (25.0)		3 (33.3)	6 (66.7)	
Widowed	2 (50.0)	2 (50.0)		4 (80.0)	1 (20.0)	
**Planned Marriage**						
Yes	68 (54.8)	56 (45.2)	χ ^2^ =7.383	52 (41.3)	74 (58.7)	χ ^2^ = 17.139
No	3 (18.8)	13 (81.3)	P=0.008*	2 (5.3)	36 (94.7)	P=<0.001*
**Type of Marriage**						
Monogamy	51 (42.1)	70 (57.9)	χ ^2^ = 6.046	56 (34.4)	107 (65.6)	χ ^2^ = 0.151
Polygamy	23 (65.7)	12 (34.3)	P=0.014*	6 (30.0)	14 (70.0)	p = 0.698

*Statistically significant N- naira (unit of currency) N- this will be used hence forth.

### Logistic regression analysis of the level of SRHC by independent variables

On regression analysis [Table 8], planned marriage (rural parents) and age, education, occupation (urban parents) did not remain significantly associated with the level of SRHC. Female rural adolescents were 1.9 times more likely to have good SRHC compared to their male counterparts while early adolescents in the urban area were 2.2 times more likely to have good SRHC compared to late adolescents in the same area.

**Table 8:** Predictors of SRHC level among respondents by their residence.

Variables	Odds Ratio	95% CI (lower- Upper)	P-values
**Adolescents**			
**Sex (Rural)**			
Male (ref)			
Female	1.912	1.053 – 3.471	0.033
**Age group (Urban)**			
10 – 14	2.218	1.215 – 4.048	0.009
15 -19 (ref)			
**Parents**			
**Rural Parents**			
**Type of Marriage**			
Monogamy Polygamy (ref)	2.346	1.022 – 5.381	0.044
**Income**			
< ₦20,000 (ref)			
₦20,000-₦50,000	1.766	0.811 – 3.844	0.152
> ₦50,000	4.920	1.284 – 18.852	0.020
**Urban Parents**			
**Sex**			
Male (ref)			
Female	3.445	1.409 – 8.422	0.007
**Planned Marriage**			
Yes (ref)	4.665	1.521 – 14.308	0.007
No			
**Length**			
≤ 30 minutes (ref)			
> 30 minutes	2.414	1.139 – 5.119	0.022
**Income**			
< ₦20,000 (ref)			
₦20,000-₦50,000	5.059	2.182 – 11.732	0.000
> ₦50,000	1.376	0.506 – 3.745	0.532

Rural parents in monogamous marriage and those who earn above ₦50,000 were 2.3 times and about 5 times more likely to have good level of SRHC with their adolescents than those in polygamous marriage and who earn less than ₦20,000, respectively. Urban parents in unplanned marriage were nearly 5 times more likely to have good SRHC than those whose marriages were planned, and mothers were 3.4 times more likely than fathers to have good SRHC with their adolescents. SRH discussions with urban parents that lasted more than 30 minutes were twice as likely to be more beneficial than those that lasted less than or equal to 30 minutes. Additionally, those who earn between ₦20,000 and ₦50,000 were five times more likely to have good SRHC than those who earn less than ₦20,000.

## Discussion

This study evaluated SRHC between parents and their in-school adolescents in rural and urban areas of Osun State to identify key similarities, disparities, and factors influencing SRHC. Unlike many previous studies that concentrated on either parents or adolescents, our research adopts a dyadic approach, exploring the interactions between parents (fathers and mothers) and adolescents (sons and daughters). Additionally, we provide a rural/urban comparison, offering insights into how the dynamics of SRHC vary between these distinct settings.

Adolescents reported a higher SRHC prevalence than parents (69.3% vs 55.5%) based on a single question (Ever discussed issues about SRH). This prevalence is lower than SRHC reported by parents in studies conducted in Ghana and northern Nigeria but comparable to adolescent SRHC prevalence in studies conducted in Osun State [25], north Nigeria [15], and Ghana [14]. The reason for adolescents reported higher SRHC prevalence compared to their parents is unclear. Still, it may be due to more consciousness of SRHC on the part of the adolescents, with more parents being forgetful about such discussions.

Some parents in this study had never had SRH discussions with their adolescents, and the primary reason for this is that their children were still too young for SRH discussions. This finding is contrary to a study that reported that the majority of respondents cited a lack of knowledge about RH issues and difficulties bringing up the topic because of fear [7]. This perception by parents in our study could have severe consequences for their adolescents, who may choose to rely on their peers, social media, and strangers for information and support for their SRH needs and consequently make poor SRH choices [26]. SRH information from peers and strangers could also be fraught with misinformation about SRH issues and changes in adolescence [27]. Surprisingly, more urban parents significantly reported this perception compared to rural parents. School attendance and length of stay in school are higher in urban areas than in Nigeria’s rural areas [28].

Consequently, urban populations are more likely to delay marriage and childbirth [29]. This might be the projection of these parents who believe that their adolescents are too young for SRH discussion. However, participation in risky sexual behaviour and early sexual debut cuts across adolescents in both urban and rural areas [30].

This study revealed that some fathers believed that SRH discussion is the responsibility of mothers. Our findings parallel a Uganda study that reported that fathers abdicate their roles of communicating about SRH to mothers [31]. This perspective was more strongly held in urban areas than rural areas. It may be linked to the fast-paced environment of the metropolitan regions where parents, particularly fathers, are tied up outside the home for extended periods due to work schedules and pressure [32]. This work-related stressor leaves little room for SRHC between parents and their adolescents, which exposes adolescents to reliance on incorrect or false SRH information through alternative sources [27]. Some parents in both our study locations, with a higher proportion among parents in rural areas, were hesitant to talk to their adolescents about sexual matters out of concern that it could facilitate indulging in sexual practices. This finding is consistent with a study in Harar, Eastern Ethiopia [7].

SRHC was generally higher among females than males and with early adolescents (10-14 years) than late adolescents in both locations. This difference was significantly higher for females in the rural area and early adolescents in the urban area. This agrees with earlier studies done in the USA, West Ethiopia, and Zambia in which parents tend to communicate less with their adolescents as they advance in age [33–35]. Furthermore, mothers talked to their children more than the fathers in both study locations, comparable to studies conducted in Nigeria, Ghana, and Tanzania [14,25,36] but contrary to an Ethiopian survey that found that fathers communicated more with their teenage children [7]. However, our findings on higher levels of SRHC by fathers in rural areas compared to their urban counterparts are consistent with the Ethiopian study. A possible explanation is that living in rural communities is associated with less work pressure and stress, allowing for more family time, cohesion, and discussion.

Though several respondents, especially the adolescents, reported a regular schedule and length of SRHC in the rural and urban areas, assessment of the level of SRHC, as determined by the 20 sexual topics, showed that the majority of the adolescents reported a poor level of SRHC. In contrast, the majority of the parents rated their level of SRHC as good. The level of SRHC was significantly higher among urban parents compared to their rural counterparts. This suggests that discussions around these topics held between parents and their adolescents were perceived as sufficient by the parents, while their adolescents perceived them as inadequate. The reasons for this disparity might be the need for more depth whenever the topics are discussed and different perceptions of SRHC such that adolescents cannot recognize and acknowledge such discussions as they are often devoid of meaningful dialogues. This is in line with previous studies where parents were adjudged to speak in parables when communicating about sexuality [15,37]. Their messages need to be delivered more transparently and understandably.

Our findings also showed that more parents shied away from discussing topics around experiencing sex compared to other SRH topic domains. This is not surprising because it has been documented that explicit discussion of sex with their children is often problematic for parents, and more often, they resort to the use of euphemisms that further confuse their adolescents [26,27,31]. Whereas there are available and accessible factual print and online materials on the biological and sexual risk prevention domains, materials on experiencing sex are scarce [38,39]. Expanding the scope of parent-adolescent SRHC to include the topic domain on experiencing sex is pertinent because it will assist and reinforce the adolescents in making guided and informed SRH decisions. SRHC materials specific to SRH topics such as sexual feelings, choice of sexual partners, rape, pornography, and sexual debut should be developed and accessible to both parents and adolescents.

Our study has some limitations. First, it was a cross-sectional survey; hence, a cause-effect relationship could not be established. Second, it was conducted among only in-school adolescents and their parents in selected schools across only six LGAs and based on participants’ self-reports; hence, caution should be exercised in generalizing the results. However, we employed a mixed data collection method among dyads of parents and their adolescents in both rural and urban areas, which allowed an in-depth and comparable exploration of SRHC. Furthermore, we used a multistage sampling to select participants across diverse geo-political zones, LGAs, and locations. The findings highlight potential entry points for interventions in parents-adolescents SRHC.

## Conclusion

Although many parents in this study engage in SRHC with their adolescents, such discussions were inadequate and sometimes reactive and sporadic, triggered by suspicion of sexual activity. Fathers were still less involved in SRHC, and the male adolescents were less communicated with than the females. Reasons for non-discussion by some parents highlighted their perception and attitude towards SRHC. In addition, essential topics relating to experiencing sex are often not discussed, and the style of SRHC was ambiguous. These might be inadequate to protect and facilitate healthy SRH choices among adolescents. Hence, parents need to be enlightened about the importance, depth, and practical approaches toward SRHC. Knowledge and self-efficacy building activities will equip parents and adolescents with the skills required for early, effective, and sustained engagement on SRH issues, allowing for a “talk-the-talk” before the adolescents “walk the walk,” thereby ensuring adolescents live up to their most significant SRH potential and right. Health policymakers and program planners should ensure the inclusion of detailed and accurate SRH information covering all three domains of sexual topics (biological/developmental, sexual risk prevention, and experiencing sex) in the promotion of SRHC in school curricula and general public health enlightenment programmes. Bridging the resource and knowledge gap surrounding the underexplored SRH topic domain of experiencing sex requires dedicated efforts. Educational initiatives, research, and community outreach programs should be designed to provide accurate information, dispel myths, and create a supportive environment for discussing and understanding the complexities of sexual experiences between parents and their adolescents.

## Supporting information

S1 Table LGAs in Osun State with categorization into rural and urban. (PDF)

S1 Questionnaire Study questionnaire for parents and adolescents. (DOCX)

S1 Data Study data on adolescents. (SAV)

S2 Data Study data on parents. (SAV)
