# Supplementary material for: Magnitude of sexual and reproductive health communication between parents and their adolescents: Evidence from Osun State, Nigeria
Source: PLOS Glob Public Health. 2025 Feb 10;5(2):e0004034. doi: 10.1371/journal.pgph.0004034 (PMC11809892; doi:10.1371/journal.pgph.0004034)
Supplement: S1 Table — (PDF) [file pgph.0004034.s001.pdf]

S1Table : LGAs in Osun State with categorization into rural and urban.

SENATORIAL DISTRICTS					
**Osun Central**		**Osun East**		**Osun West**	
**Rural**	**Urban**	**Rural**	**Urban**	**Rural**	**Urban**
Boripe	Ifelodun	Obokun	Ife East	Egbedore	Ayedaade
Ifedayo	Irepodun	Atakumosa West	Ilesa West	Ejigbo	Ede North
Odo-Otin	Olorunda	Ife North	Ilesha East	Ola-Oluwa	Ede South
Orolu	Osogbo	Ife South	Ife Central	Isokan	Iwo
Boluwaduro	Ila	Oriade		Ayedire	
		Atakumosa East		Irewole	

Source: National Population Commission, Nigeria. Nigeria Population Census 1991.
