# Supplementary material for: Magnitude of sexual and reproductive health communication between parents and their adolescents: Evidence from Osun State, Nigeria
Source: PLOS Glob Public Health. 2025 Feb 10;5(2):e0004034. doi: 10.1371/journal.pgph.0004034 (PMC11809892; doi:10.1371/journal.pgph.0004034)
Supplement: S1 Questionnaire — (DOCX) [file pgph.0004034.s002.docx]

**ADOLESCENT QUESTIONNAIRE**

**Comparative Study of Sexual and Reproductive Health Communication between Parents and their Adolescents in Rural and Urban Areas of Osun State, Nigeria.**

I am a community Health Physician in training, working in the Department of Community Health of LAUTECH Teaching Hospital, Osogbo. I am currently conducting a study on Sexual and Reproductive Health (SRH) Communication among parents and adolescents in Osun State. The purpose of this study is to gain an in-depth understanding of communication between parents/guardian and their in-school adolescents on SRH issues.

Your identity, responses and opinions will be kept strictly confidential and will be used for the purpose of this research only. Please ask if you don’t understand any question.

Thank you.

Date ………….. Questionnaire ID: ……… LGA …………….

**SECTION A: Socio-Demographic Characteristics**

Please fill or tick appropriate responses in the spaces provided

1. Name of school_________________
2. Class____________________
3. Age as at last birthday (in years) _________________________
4. Sex: (1) Male [ ] (2) Female [ ]
5. Ethnicity: (1) Igbo [ ] (2) Yoruba [ ] (3) Hausa [ ] (4) Others ___________
6. Who do you currently live with? (1) Both parents [ ] (2) Mother only [ ]

(3) Father only [ ] (4) Male Guardian [ ] (5) Female Guardian [ ]

1. Religion:(1) Christianity [ ] (2) Islam [ ] (3) Traditional [ ] (4) Others specify _____
2. Birth position: ______________________
3. Family status: How many brothers and sisters do you have? __________________
4. Father’s level of education: (1) Primary [ ] (2) Secondary [ ] (3) Tertiary [ ] (4) None [ ] (5) Others (please specify) _____________
5. Mother’s level of Education: (1) Primary [ ] (2) Secondary [ ] (3) Tertiary [ ] (4) None [ ] (5) Others (please specify) _____________
6. Father’s Occupation: (1) Civil servant [ ] (2) Farmer [ ] (3) Businessman/Trader [ ] (4) Unemployed [ ] (5) Others (please specify) _____________
7. Mother’s Occupation: (1) Civil servant [ ] (2) Farmer [ ] (3) Businesswoman/Trader [ ] (4) Unemployed [ ] (5) Others (please specify)_____________

**SECTION B: Knowledge of Adolescent Sexual and Reproductive Health**

1. Have you ever heard of sexual and reproductive health issues?
2. Yes [ ] (2) No [ ] If No, end the interview.
3. Where did you hear about sexual and reproductive health issues? (please tick all that applies) (1) Mass media [ ] (2) School [ ] (3) Parents/Guardian [ ] (4) Friends [ ] (5) Other relatives [ ] (6) Others (specify)…………………..

| S/N | Questions | Yes | No | Not sure |
| --- | --- | --- | --- | --- |
|  | Components of adolescent sexual and reproductive health are: |  |  |  |
|  | Changes at puberty |  |  |  |
|  | Teenage pregnancy |  |  |  |
|  | Sexually Transmitted Infection / HIV |  |  |  |
|  | Unsafe abortion |  |  |  |
|  | Family planning |  |  |  |
|  | Signs of sexual maturity in adolescents are: |  |  |  |
|  | Wet dreams |  |  |  |
|  | Menstruation |  |  |  |
|  | Enlargement of breasts |  |  |  |
|  | Deepening of voice |  |  |  |
|  | Signs of Sexually Transmitted Infections are: |  |  |  |
|  | Ulcers and sores around the genitals |  |  |  |
|  | Painful intercourse |  |  |  |
|  | Painful urination |  |  |  |
|  | Discharge of blood from the genitals |  |  |  |
|  | A girl can get pregnant at first sexual experience |  |  |  |

1. List three characteristics associated with puberty in adolescents

(1)………………………………… (2)……………………………

(3)…………………………………

1. Mention 2 methods that can be used to prevent involvement of adolescent in risky sexual behaviour

(1)………………………………………………… (2)…………………………………………………

1. Mention three common sexual and reproductive health infections/diseases among adolescents

(1)……………………… (2)……………………… (3)………………………

1. Mention two ways by which adolescents can contract HIV/AIDS and other sexually transmitted infections

(1)………………………………… (2) ………………………………

1. List two methods of preventing transmission of HIV/AIDS and other sexually transmitted diseases in adolescents

(1)………………………………… (2)……………………………….

1. Mention 3 methods that can be used to prevent unwanted pregnancy

(1)…………………………………………… (2)……………………………………………

(3)……………………………………………

**Section C: Prevalence and Pattern of Parental communication of sexual and reproductive health messages with adolescents**

1. Has your parent/guardian ever discussed issues relating to sexual and reproductive health with you? (1) Yes [ ] (2) No [ ] If no, go to question 40
2. How often do you discuss issues about sexual and reproductive health?

(1) Daily [ ] (2) Weekly [ ] (3) Monthly [ ] (4) Yearly [ ]

(5) Whenever I’m noticed to be engaging in activities that relate to sexuality[ ]

(6) No specific interval [ ] (7) Others (specify) …………………….

1. How many minutes does the discussion usually take on the average?

(1) Less than 10 minutes [ ] (2) 11 – 20 minutes [ ] (3) 21 – 30 minutes [ ] (4) 31 – 1 hour [ ] (5) More than 1 hour [ ] (6) Not specific [ ]

1. Can you say that the discussion your parents/guardian had with you on issues relating to sexual and reproductive health is adequate? (1) Yes [ ] (2) No [ ]
2. How old were you when your parent/guardian started discussion on sexual and reproductive health issues with you? ______________
3. Using a scale from 0 to 3, with 0 = none and 3 = a lot, please indicate (tick) how much discussion you have had with your **Father/Male Guardian** about the following topic

|  | **Category** | **Topics** | 0 | 1 | 2 | 3 |
| --- | --- | --- | --- | --- | --- | --- |
|  | Development/biological aspect | Physical development (function and structure) |  |  |  |  |
|  |  | Menstruation/wet dreams |  |  |  |  |
|  |  | Puberty |  |  |  |  |
|  |  | Masturbation |  |  |  |  |
|  |  | Reproduction /having babies |  |  |  |  |
|  | Sexual risk prevention | Prevention of Sexually transmitted disease |  |  |  |  |
|  |  | Prevention of HIV/AIDS |  |  |  |  |
|  |  | Abstaining from sex until marriage |  |  |  |  |
|  |  | Use of condom & other contraceptives |  |  |  |  |
|  |  | Pregnancy |  |  |  |  |
|  |  | Abortion |  |  |  |  |
|  |  | Consequences of premarital sex |  |  |  |  |
|  |  | Substance use |  |  |  |  |
|  | Experiencing sex | Sexual feeling |  |  |  |  |
|  |  | When to start sexual intercourse |  |  |  |  |
|  |  | Choosing sexual partners |  |  |  |  |
|  |  | How to handle sexual pressure |  |  |  |  |
|  |  | Homosexuality |  |  |  |  |
|  |  | Rape |  |  |  |  |
|  |  | Pornography |  |  |  |  |

1. Using a scale from 0 to 3, with 0 = none and 3 = a lot, please indicate (tick) how much discussion you have had with your **Mother/Female Guardian** about the following topics

|  | **Category** | **Topics** | 0 | 1 | 2 | 3 |
| --- | --- | --- | --- | --- | --- | --- |
|  | Development/biological aspect | Physical development (function and structure) |  |  |  |  |
|  |  | Menstruation/wet dreams |  |  |  |  |
|  |  | Puberty |  |  |  |  |
|  |  | Masturbation |  |  |  |  |
|  |  | Reproduction /having babies |  |  |  |  |
|  | Sexual risk prevention | Prevention of Sexually transmitted disease |  |  |  |  |
|  |  | Prevention of HIV/AIDS |  |  |  |  |
|  |  | Abstaining from sex until marriage |  |  |  |  |
|  |  | Use of condom & other contraceptives |  |  |  |  |
|  |  | Pregnancy |  |  |  |  |
|  |  | Abortion |  |  |  |  |
|  |  | Consequences of premarital sex |  |  |  |  |
|  |  | Substance use |  |  |  |  |
|  | Experiencing sex | Sexual feeling |  |  |  |  |
|  |  | When to start sexual intercourse |  |  |  |  |
|  |  | Choosing sexual partners |  |  |  |  |
|  |  | How to handle sexual pressure |  |  |  |  |
|  |  | Homosexuality |  |  |  |  |
|  |  | Rape |  |  |  |  |
|  |  | Pornography |  |  |  |  |

1. Overall, which of this best describe your parent’s/guardian’s level of communication on sexual and reproductive health issues with you?

(1) Low [ ] (2) Moderate [ ] (3) High [ ]

**SECTION D: Methods Parents Adopt in Communicating Sexual and Reproductive Health Issues**

1. What informs your parent’s/guardian’s discussion on sexuality with you? (you can tick more than one response) (1) I asked questions [ ] (2) T.V./Radio [ ]

(3) Bad experiences of other people [ ] (4) Parent/guardian initiative [ ]

(5) I was caught with pornographic materials [ ] (6) Parent/guardian suspected me of sexual activity (7) Others (specify)……..

1. What method did your parent/guardian adopt in discussing issues relating to sexual and reproductive health with you? (you can tick more than one response)

(1) Give me text based materials like books and magazines [ ]

(2) Expose me to health programmes on Television or Radio [ ]

(3) Make me to watch movies/films on sexual and reproductive health [ ]

(4) One on one discussion [ ] (5) General family discussion [ ]

(6) Others (specify)………………….

**PARENT QUESTIONNAIRE**

“**Comparative Study of Sexual and Reproductive Health Communication between Parents and their Adolescents in Rural and Urban Areas of Osun State, Nigeria.”**

I am a community Health Physician in training, working in the Department of Community Health of LAUTECH Teaching Hospital, Osogbo. I am currently conducting a study on Sexual and Reproductive Health (SRH) Communication among parents and adolescents in Osun State. The purpose of this study is to gain an in-depth understanding of communication between parents and their in-school adolescents on SRH issues.

Your identity, responses and opinions will be kept strictly confidential and will be used for the purpose of this research only.

Thanks.

Date ………….. Questionnaire ID: ……… LGA …………

**SECTION A: Socio- Demographic Characteristics**

**Instruction: Please respond to the following questions;**

1. Age as at last birthday (in years) -------------------------
2. Sex: (1) Male [ ] (2) Female [ ]
3. Level of education attained: (1) Primary [ ] (2) Secondary [ ] (3) Tertiary [ ] (4) None [ ] Others (please specify)……………
4. Ethnicity: (1) Igbo [ ] (2) Yoruba [ ] (3) Hausa [ ] (4) Others (please specify)…………
5. Religion: (1) Christianity [ ] (2) Islam [ ] (3) Traditional [ ]

(4) Others (please specify) …………..

1. Marital status: (1) Single [ ] (2) Married [ ] (3) Cohabiting [ ]

(4) Divorced [ ] (5) Separated [ ] (6) Widowed [ ]

1. Type of marriage: (1) Monogamy [ ] (2) Polygamy [ ]
2. Age at which you married (in years) ……………………
3. Was your marriage planned? (1) Yes [ ] (2) No [ ]
4. Duration of marriage (in years).....................
5. Number of children ……………..
6. Occupation: (1) Civil servant [ ] (2) Farmer [ ] (3) Business man//Trader [ ] (4) Unemployed (5) Others (please specify)………………
7. Average monthly Income (in Naira)...........................

**SECTION B: Knowledge of Adolescent Sexual and Reproductive Health** (please tick as appropriate)

| S/N | Questions | Yes | No | Not sure |
| --- | --- | --- | --- | --- |
| 14 | Components of adolescent sexual and reproductive health are: |  |  |  |
|  | Changes at puberty |  |  |  |
|  | Teenage pregnancy |  |  |  |
|  | STI/HIV |  |  |  |
|  | Unsafe abortion |  |  |  |
|  | Family planning |  |  |  |
| 15 | Signs of sexual maturity in adolescents are: |  |  |  |
|  | Wet dreams |  |  |  |
|  | Menstruation |  |  |  |
|  | Enlargement of breasts |  |  |  |
|  | Deepening of voice |  |  |  |
| 16 | Signs of Sexually Transmitted Infections are: |  |  |  |
|  | Ulcers and sores around the genitals |  |  |  |
|  | Painful intercourse |  |  |  |
|  | Painful urination |  |  |  |
|  | Discharge of blood from the genitals |  |  |  |
| 17 | A girl can get pregnant at first sexual experience |  |  |  |

18. List three characteristics associated with puberty in adolescents

(1)………………………………… (2)……………………………

(3)…………………………………

19. Mention 2 methods that can be used to prevent involvement of adolescents in risky sexual behaviour

(1)…………………………………………………….(2)…………………………………

20. Mention three common sexual and reproductive health infections/diseases among adolescents

(1)……………………… (2)……………………… (3)………………………

21. Mention two ways by which male adolescents can contract HIV/AIDS and other sexually transmitted infections

(1)………………………………… (2) ………………………………

22. List two methods of preventing transmission of HIV/AIDS and other sexually transmitted diseases

(1)………………………………… (2)……………………………….

**SECTION C: Magnitude and Pattern of Parental communication of sexual and reproductive health messages with adolescents**

23. Have you ever discussed issues relating to sexual and reproductive health with your child/ward? (1) Yes [ ] (2) No [ ]

**If No, answer question 24, then skip to question 37**

24. Why have you not discussed issues relating to sexual and reproductive health with your child/ward? ---------------------------------------------------------------------------------

25. How often do you discuss issues about sexual and reproductive health?

(1) Daily [ ] (2) Weekly [ ] (3) Monthly [ ] (4) Yearly [ ]

(5) Whenever I notice him engaging in activities that relate to sexuality [ ]

(6) No specific interval [ ] (7) Others (specify) …………………….

26. How many minutes does the discussion usually take on the average?

(1) Less than 10 minutes [ ] (2) 11 – 20 minutes [ ] (3) 21 – 30minutes [ ] (4) 31 – 1 hour [ ] (5) More than 1 hour [ ] (6) Not specific [ ]

27. Can you say that the discussion you have on issues relating to sexual and reproductive health with adolescent is adequate? (1) Yes [ ] (2) No [ ]

28. How old was/were your child (ren)/ward when you started discussion on sexual and reproductive health? …………….

29. Using a scale from 0 to 3, with 0 = none and 3 = a lot, please indicate (circle) how much discussion you have had with your adolescent child/ward about the following topics

|  | **Category** | **Topics** | 0 | 1 | 2 | 3 |
| --- | --- | --- | --- | --- | --- | --- |
|  | Development/biological aspect | Physical development (function and structure) |  |  |  |  |
|  |  | Menstruation/wet dreams |  |  |  |  |
|  |  | Puberty |  |  |  |  |
|  |  | Masturbation |  |  |  |  |
|  |  | Reproduction /having babies |  |  |  |  |
|  | Sexual risk prevention | Prevention of Sexually transmitted disease |  |  |  |  |
|  |  | Prevention of HIV/AIDS |  |  |  |  |
|  |  | Abstaining from sex until marriage |  |  |  |  |
|  |  | Use of condom & other contraceptives |  |  |  |  |
|  |  | Pregnancy |  |  |  |  |
|  |  | Abortion |  |  |  |  |
|  |  | Consequences of premarital sex |  |  |  |  |
|  |  | Substance use |  |  |  |  |
|  | Experiencing sex | Sexual feeling |  |  |  |  |
|  |  | When to start sexual intercourse |  |  |  |  |
|  |  | Choosing sexual partners |  |  |  |  |
|  |  | How to handle sexual pressure |  |  |  |  |
|  |  | Homosexuality |  |  |  |  |
|  |  | Rape |  |  |  |  |
|  |  | Pornography |  |  |  |  |

30. Overall, which of this best describe your level of communication on sexual and reproductive health issues with your adolescent child?

(1) Low [ ] (2) Moderate [ ] (3) High [ ]

**SECTION D: Methods Parents Adopt in Communicating Sexual and Reproductive Health Issues**

31. What informs the discussion on sexuality with your adolescent? (Pls tick all that apply)

(1) Age of the child [ ] (2) Child asks questions [ ] (3) T.V./Radio programmes [ ]

(4) Bad experiences of other people[ ] (5) Child caught with pornographic materials[ ] (6) My initiative [ ] (7) Encouragement from religious leaders (8) Others (specify) …

32. What method did you adopt in discussing issues relating to sexual and reproductive health with your adolescent child? (Please tick all that apply)

(1) Give my child text based materials like books and magazines [ ]

(2) Expose my child to health programmes on Television or Radio [ ]

(3) Make my child to watch movies/films on sexual and reproductive health [ ]

(4) One on one discussion [ ] (5) General family discussion [ ]

(6) Others (specify)………………….
